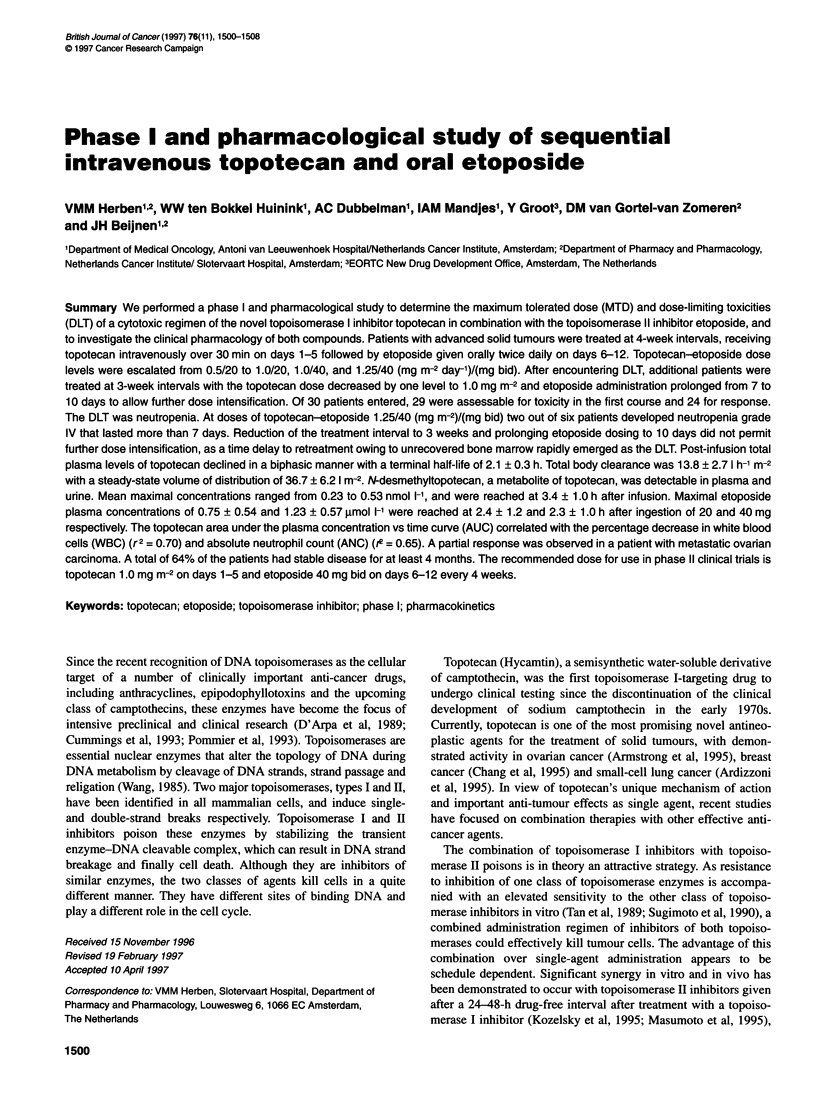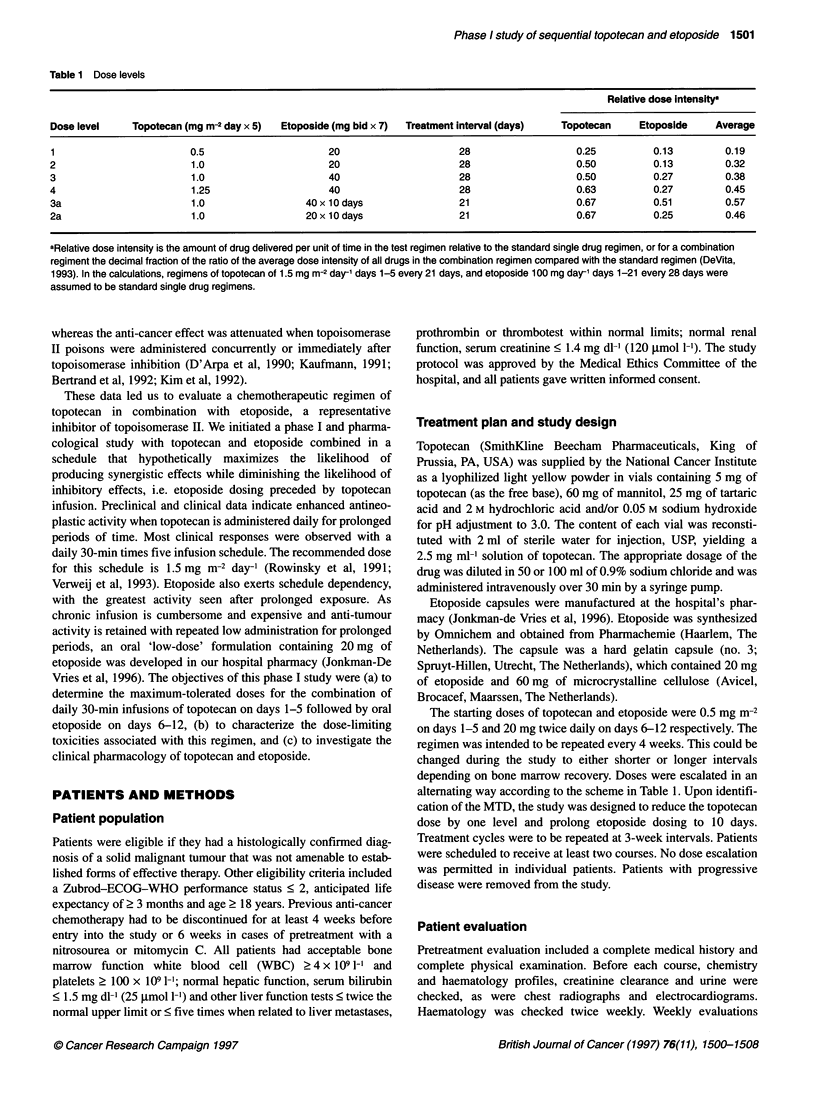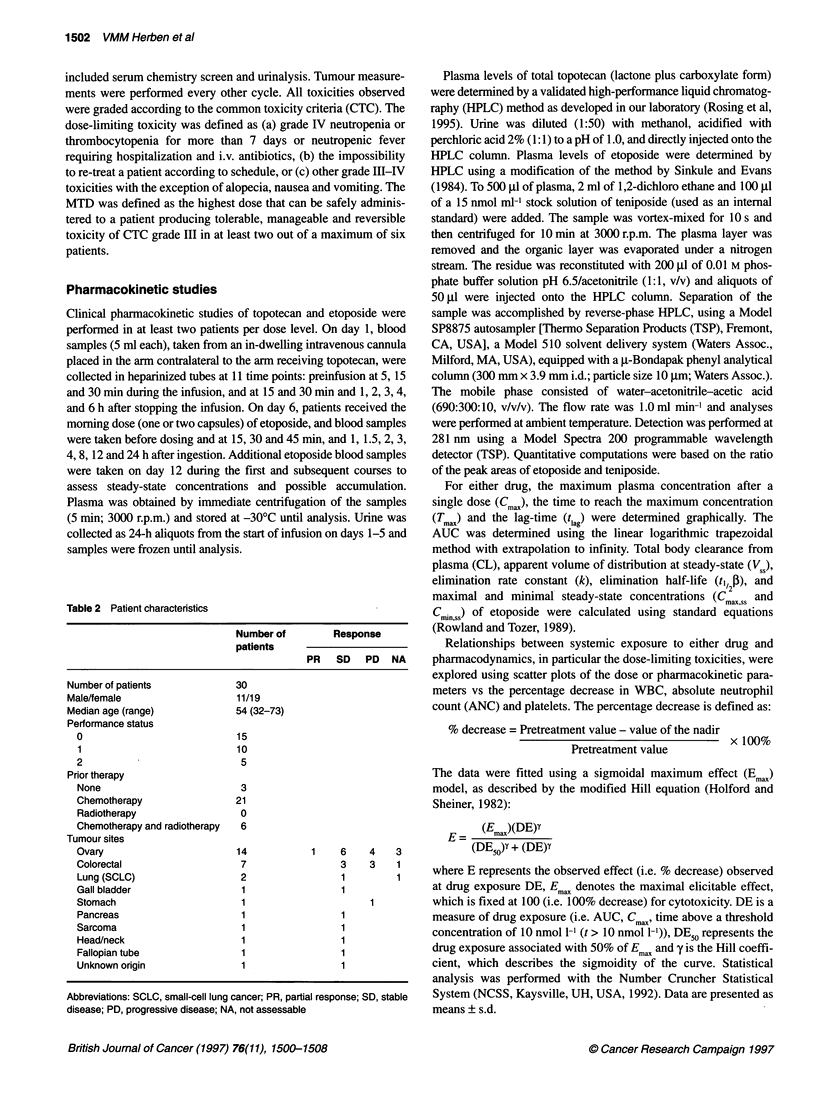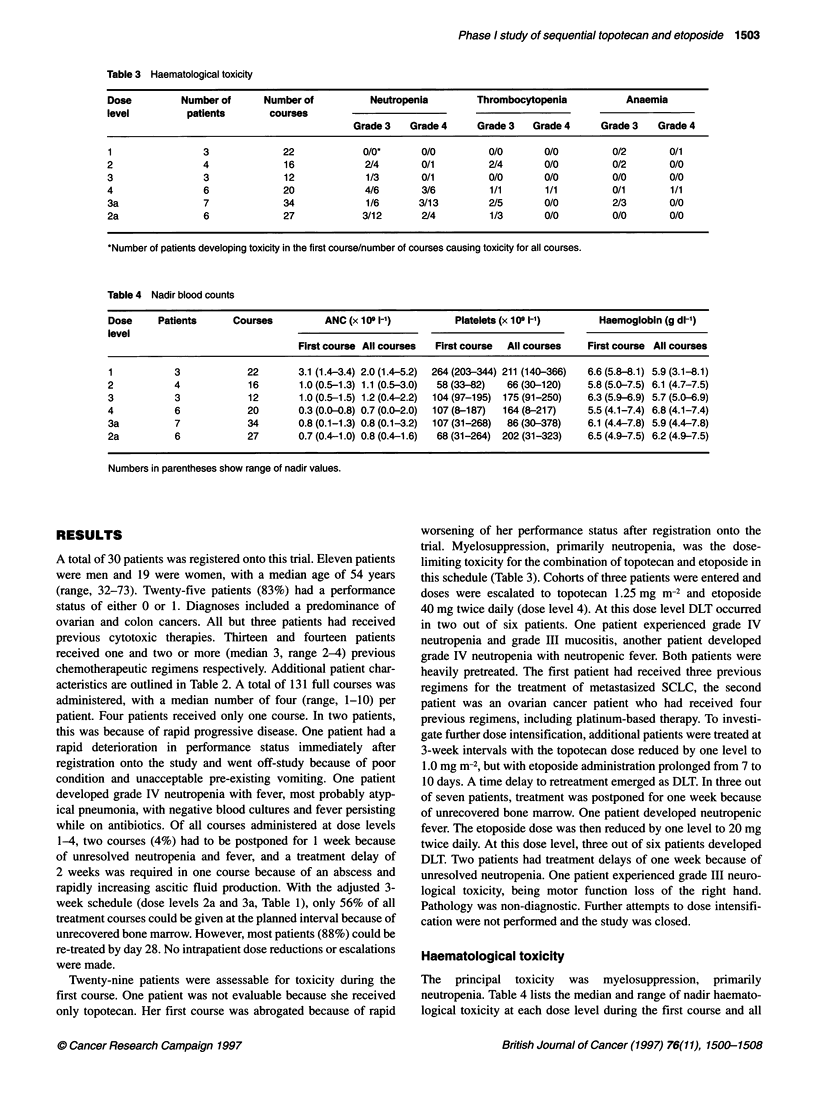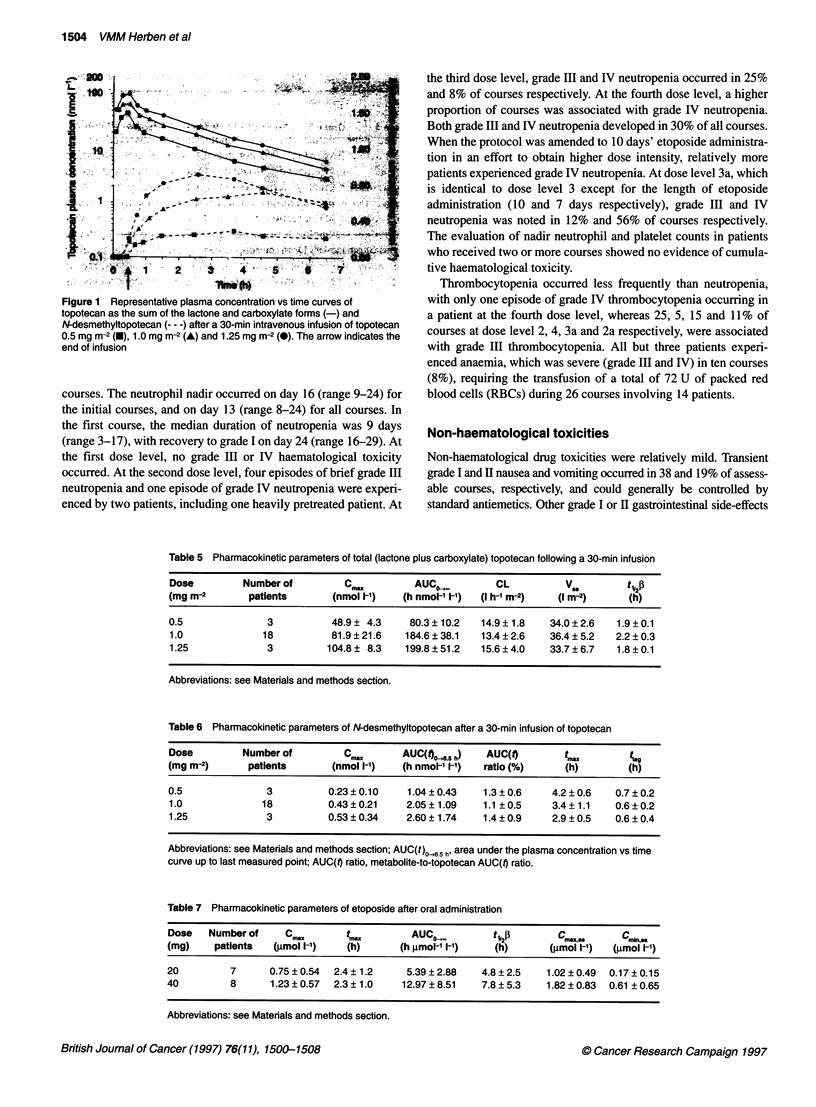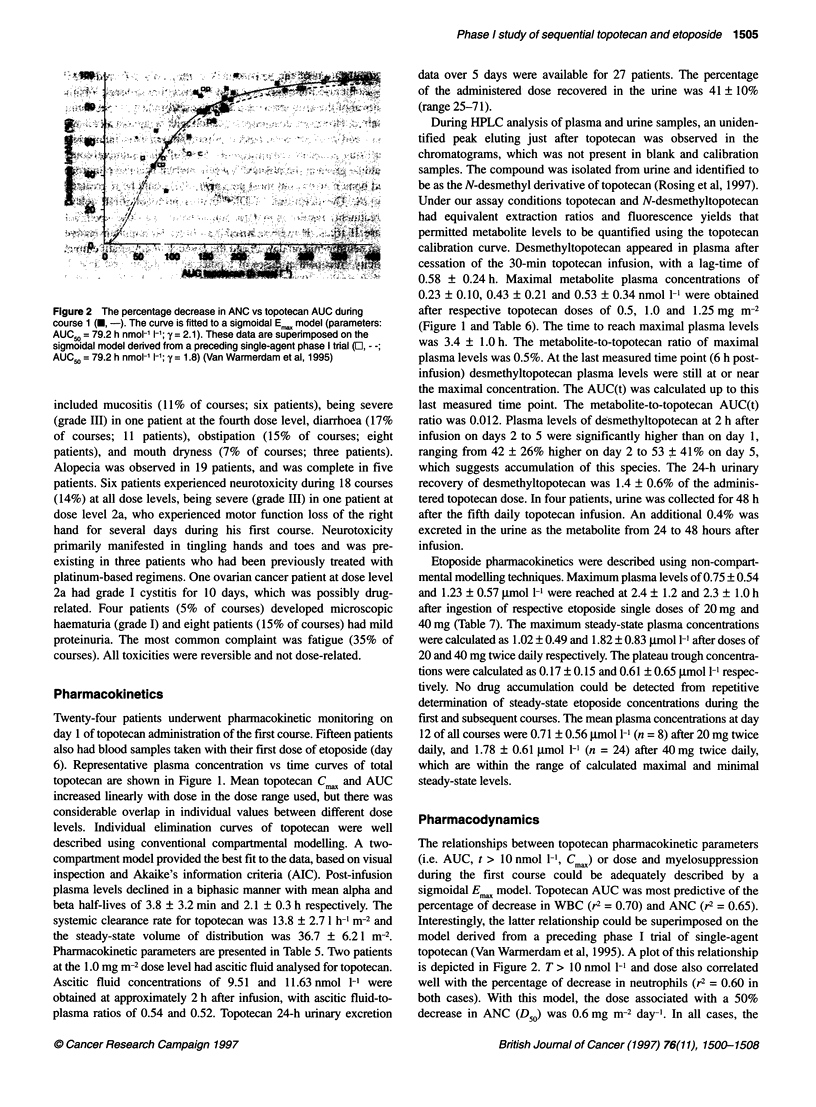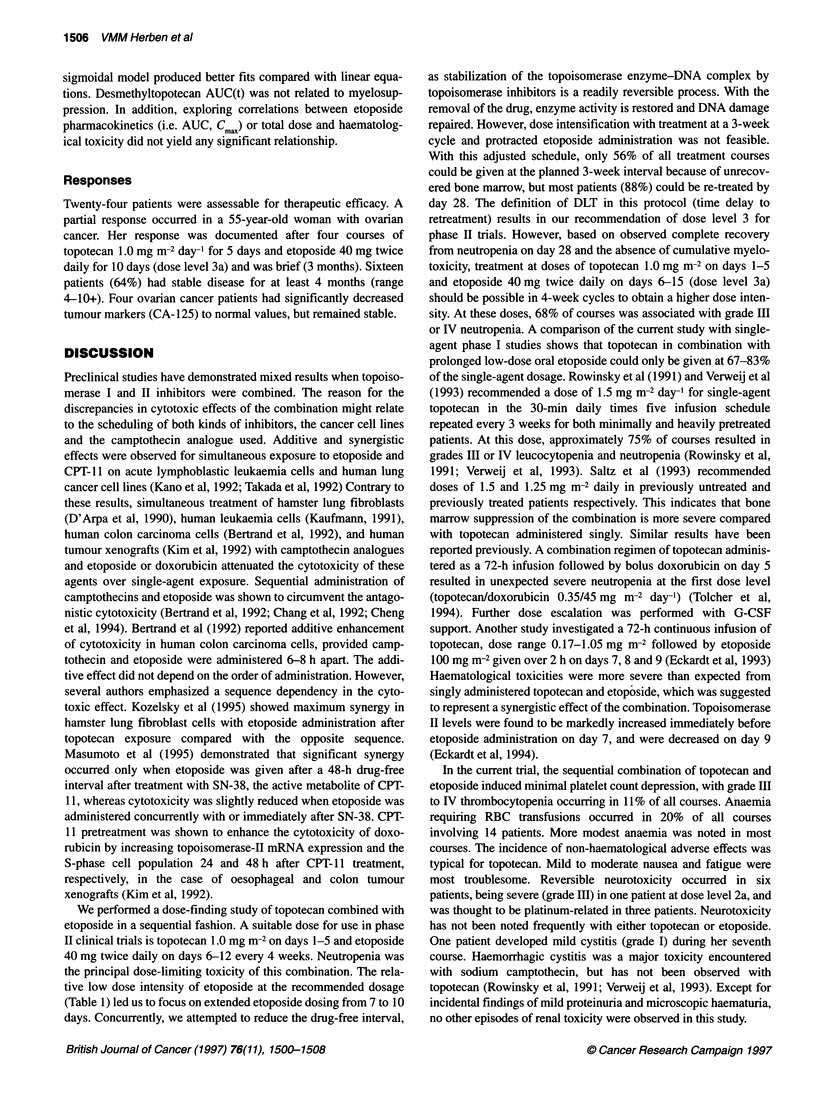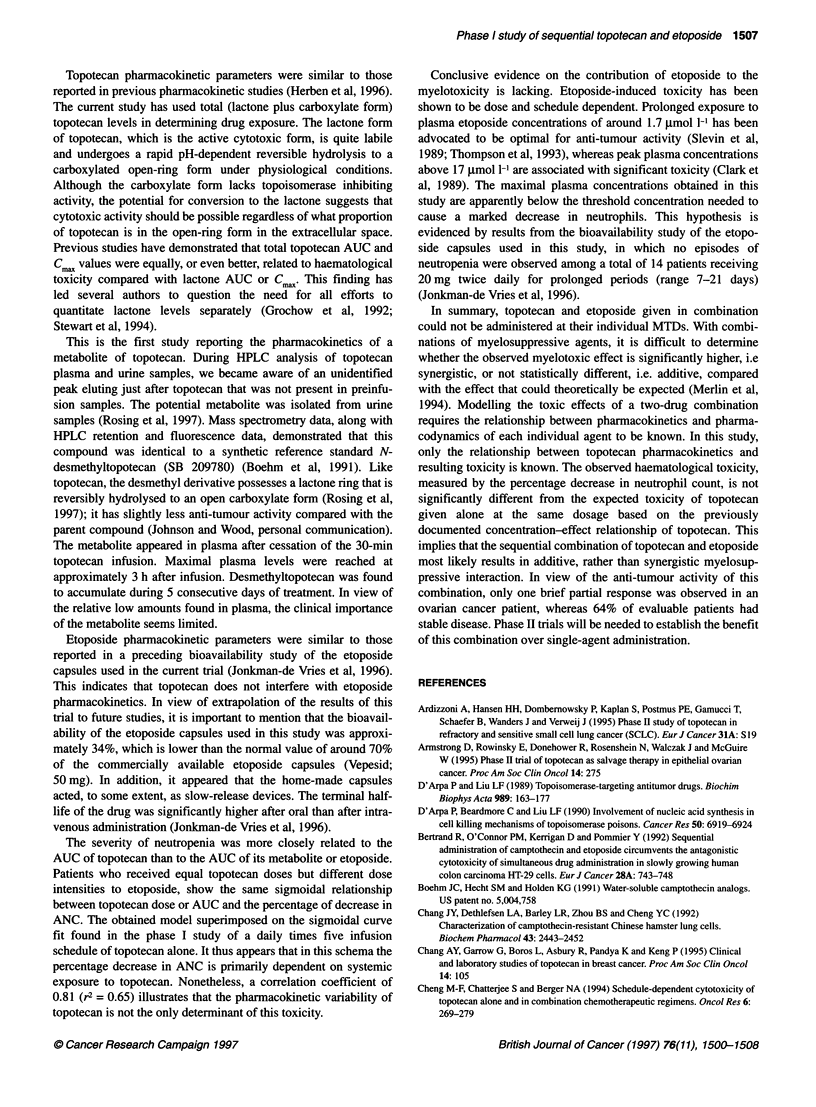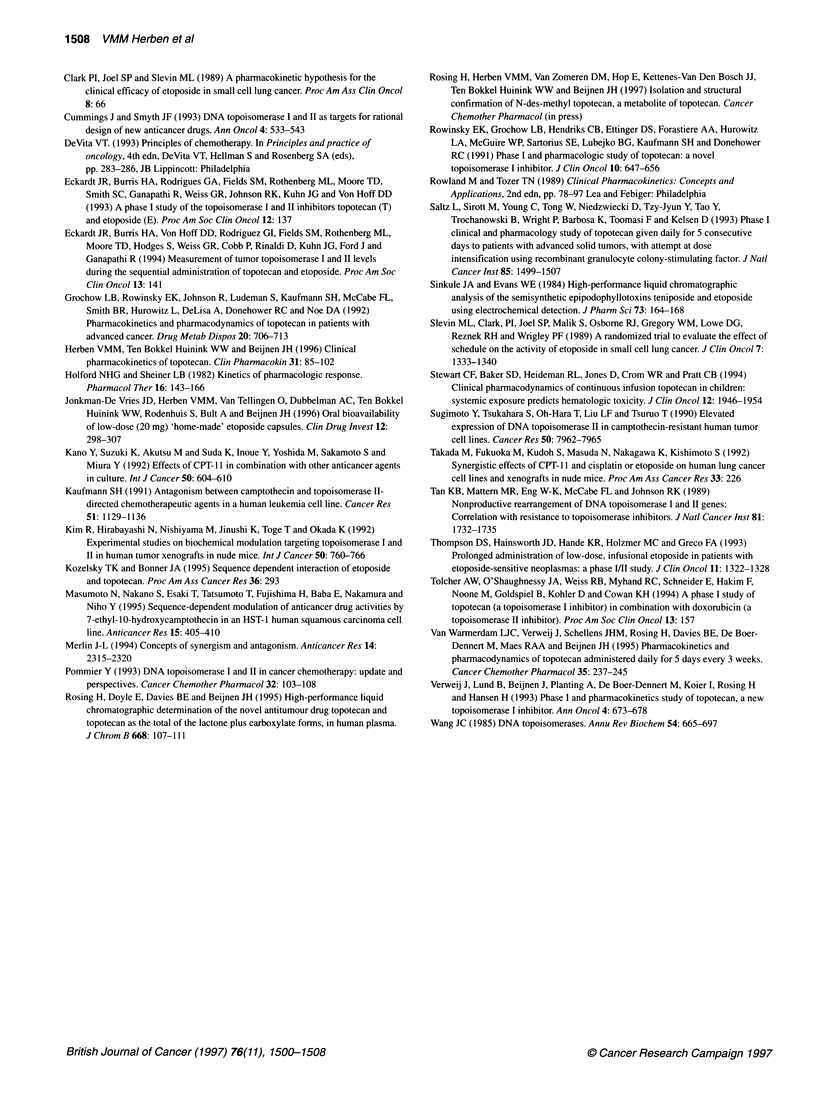# Phase I and pharmacological study of sequential intravenous topotecan and oral etoposide.

**DOI:** 10.1038/bjc.1997.585

**Published:** 1997

**Authors:** V. M. Herben, W. W. ten Bokkel Huinink, A. C. Dubbelman, I. A. Mandjes, Y. Groot, D. M. van Gortel-van Zomeren, J. H. Beijnen

**Affiliations:** Department of Medical Oncology, Antoni van Leeuwenhoek Hospital/Netherlands Cancer Institute, Amsterdam.

## Abstract

We performed a phase I and pharmacological study to determine the maximum tolerated dose (MTD) and dose-limiting toxicities (DLT) of a cytotoxic regimen of the novel topoisomerase I inhibitor topotecan in combination with the topoisomerase II inhibitor etoposide, and to investigate the clinical pharmacology of both compounds. Patients with advanced solid tumours were treated at 4-week intervals, receiving topotecan intravenously over 30 min on days 1-5 followed by etoposide given orally twice daily on days 6-12. Topotecan-etoposide dose levels were escalated from 0.5/20 to 1.0/20, 1.0/40, and 1.25/40 (mg m-2 day-1)/(mg bid). After encountering DLT, additional patients were treated at 3-week intervals with the topotecan dose decreased by one level to 1.0 mg m-2 and etoposide administration prolonged from 7 to 10 days to allow further dose intensification. Of 30 patients entered, 29 were assessable for toxicity in the first course and 24 for response. The DLT was neutropenia. At doses of topotecan-etoposide 1.25/40 (mg m-2)/(mg bid) two out of six patients developed neutropenia grade IV that lasted more than 7 days. Reduction of the treatment interval to 3 weeks and prolonging etoposide dosing to 10 days did not permit further dose intensification, as a time delay to retreatment owing to unrecovered bone marrow rapidly emerged as the DLT. Post-infusion total plasma levels of topotecan declined in a biphasic manner with a terminal half-life of 2.1 +/- 0.3 h. Total body clearance was 13.8 +/- 2.7 l h-1 m-2 with a steady-state volume of distribution of 36.7 +/- 6.2 l m-2. N-desmethyltopotecan, a metabolite of topotecan, was detectable in plasma and urine. Mean maximal concentrations ranged from 0.23 to 0.53 nmol l-1, and were reached at 3.4 +/- 1.0 h after infusion. Maximal etoposide plasma concentrations of 0.75 +/- 0.54 and 1.23 +/- 0.57 micromol l-1 were reached at 2.4 +/- 1.2 and 2.3 +/- 1.0 h after ingestion of 20 and 40 mg respectively. The topotecan area under the plasma concentration vs time curve (AUC) correlated with the percentage decrease in white blood cells (WBC) (r2 = 0.70) and absolute neutrophil count (ANC) (r2 = 0.65). A partial response was observed in a patient with metastatic ovarian carcinoma. A total of 64% of the patients had stable disease for at least 4 months. The recommended dose for use in phase II clinical trials is topotecan 1.0 mg m-2 on days 1-5 and etoposide 40 mg bid on days 6-12 every 4 weeks.


British Journal of Cancer (1997) 76(11), 1500–1508
© 1997 Cancer Research Campaign

### Phase I and pharmacological study of sequential intravenous topotecan and oral etoposide

VMM Herben[1,2], WW ten Bokkel Huinink[1], AC Dubbelman[1], IAM Mandjes[1], Y Groot[3], DM van Gortel-van Zomeren[2] and JH Beijnen[1,2]

[1]Department of Medical Oncology, Antoni van Leeuwenhoek Hospital/Netherlands Cancer Institute, Amsterdam; [2]Department of Pharmacy and Pharmacology, Netherlands Cancer Institute/ Slotervaart Hospital, Amsterdam; [3]EORTC New Drug Development Office, Amsterdam, The Netherlands

**Summary** We performed a phase I and pharmacological study to determine the maximum tolerated dose (MTD) and dose-limiting toxicities (DLT) of a cytotoxic regimen of the novel topoisomerase I inhibitor topotecan in combination with the topoisomerase II inhibitor etoposide, and to investigate the clinical pharmacology of both compounds. Patients with advanced solid tumours were treated at 4-week intervals, receiving topotecan intravenously over 30 min on days 1–5 followed by etoposide given orally twice daily on days 6–12. Topotecan–etoposide dose levels were escalated from 0.5/20 to 1.0/20, 1.0/40, and 1.25/40 (mg $m^{-2}$ $day^{-1}$)/(mg bid). After encountering DLT, additional patients were treated at 3-week intervals with the topotecan dose decreased by one level to 1.0 mg $m^{-2}$ and etoposide administration prolonged from 7 to 10 days to allow further dose intensification. Of 30 patients entered, 29 were assessable for toxicity in the first course and 24 for response. The DLT was neutropenia. At doses of topotecan–etoposide 1.25/40 (mg $m^{-2}$)/(mg bid) two out of six patients developed neutropenia grade IV that lasted more than 7 days. Reduction of the treatment interval to 3 weeks and prolonging etoposide dosing to 10 days did not permit further dose intensification, as a time delay to retreatment owing to unrecovered bone marrow rapidly emerged as the DLT. Post-infusion total plasma levels of topotecan declined in a biphasic manner with a terminal half-life of 2.1 ± 0.3 h. Total body clearance was 13.8 ± 2.7 l $h^{-1}$ $m^{-2}$ with a steady-state volume of distribution of 36.7 ± 6.2 l $m^{-2}$. *N*-desmethyltopotecan, a metabolite of topotecan, was detectable in plasma and urine. Mean maximal concentrations ranged from 0.23 to 0.53 nmol $l^{-1}$, and were reached at 3.4 ± 1.0 h after infusion. Maximal etoposide plasma concentrations of 0.75 ± 0.54 and 1.23 ± 0.57 μmol $l^{-1}$ were reached at 2.4 ± 1.2 and 2.3 ± 1.0 h after ingestion of 20 and 40 mg respectively. The topotecan area under the plasma concentration vs time curve (AUC) correlated with the percentage decrease in white blood cells (WBC) ($r^2$ = 0.70) and absolute neutrophil count (ANC) ($r^2$ = 0.65). A partial response was observed in a patient with metastatic ovarian carcinoma. A total of 64% of the patients had stable disease for at least 4 months. The recommended dose for use in phase II clinical trials is topotecan 1.0 mg $m^{-2}$ on days 1–5 and etoposide 40 mg bid on days 6–12 every 4 weeks.

**Keywords:** topotecan; etoposide; topoisomerase inhibitor; phase I; pharmacokinetics

Since the recent recognition of DNA topoisomerases as the cellular target of a number of clinically important anti-cancer drugs, including anthracyclines, epipodophyllotoxins and the upcoming class of camptothecins, these enzymes have become the focus of intensive preclinical and clinical research (D'Arpa et al, 1989; Cummings et al, 1993; Pommier et al, 1993). Topoisomerases are essential nuclear enzymes that alter the topology of DNA during DNA metabolism by cleavage of DNA strands, strand passage and religation (Wang, 1985). Two major topoisomerases, types I and II, have been identified in all mammalian cells, and induce single- and double-strand breaks respectively. Topoisomerase I and II inhibitors poison these enzymes by stabilizing the transient enzyme–DNA cleavable complex, which can result in DNA strand breakage and finally cell death. Although they are inhibitors of similar enzymes, the two classes of agents kill cells in a quite different manner. They have different sites of binding DNA and play a different role in the cell cycle.

*Received 15 November 1996*
*Revised 19 February 1997*
*Accepted 10 April 1997*

*Correspondence to:* VMM Herben, Slotervaart Hospital, Department of Pharmacy and Pharmacology, Louwesweg 6, 1066 EC Amsterdam, The Netherlands

Topotecan (Hycamtin), a semisynthetic water-soluble derivative of camptothecin, was the first topoisomerase I-targeting drug to undergo clinical testing since the discontinuation of the clinical development of sodium camptothecin in the early 1970s. Currently, topotecan is one of the most promising novel antineoplastic agents for the treatment of solid tumours, with demonstrated activity in ovarian cancer (Armstrong et al, 1995), breast cancer (Chang et al, 1995) and small-cell lung cancer (Ardizzoni et al, 1995). In view of topotecan's unique mechanism of action and important anti-tumour effects as single agent, recent studies have focused on combination therapies with other effective anti-cancer agents.

The combination of topoisomerase I inhibitors with topoisomerase II poisons is in theory an attractive strategy. As resistance to inhibition of one class of topoisomerase enzymes is accompanied with an elevated sensitivity to the other class of topoisomerase inhibitors in vitro (Tan et al, 1989; Sugimoto et al, 1990), a combined administration regimen of inhibitors of both topoisomerases could effectively kill tumour cells. The advantage of this combination over single-agent administration appears to be schedule dependent. Significant synergy in vitro and in vivo has been demonstrated to occur with topoisomerase II inhibitors given after a 24–48-h drug-free interval after treatment with a topoisomerase I inhibitor (Kozelsky et al, 1995; Masumoto et al, 1995),

**Table 1** Dose levels

| Dose level | Topotecan (mg $m^{-2}$ day × 5) | Etoposide (mg bid × 7) | Treatment interval (days) | Relative dose intensity[a] | | |
|---|---|---|---|---|---|---|
| | | | | Topotecan | Etoposide | Average |
| 1 | 0.5 | 20 | 28 | 0.25 | 0.13 | 0.19 |
| 2 | 1.0 | 20 | 28 | 0.50 | 0.13 | 0.32 |
| 3 | 1.0 | 40 | 28 | 0.50 | 0.27 | 0.38 |
| 4 | 1.25 | 40 | 28 | 0.63 | 0.27 | 0.45 |
| 3a | 1.0 | 40 × 10 days | 21 | 0.67 | 0.51 | 0.57 |
| 2a | 1.0 | 20 × 10 days | 21 | 0.67 | 0.25 | 0.46 |

[a]Relative dose intensity is the amount of drug delivered per unit of time in the test regimen relative to the standard single drug regimen, or for a combination regiment the decimal fraction of the ratio of the average dose intensity of all drugs in the combination regimen compared with the standard regimen (DeVita, 1993). In the calculations, regimens of topotecan of 1.5 mg $m^{-2}$ $day^{-1}$ days 1–5 every 21 days, and etoposide 100 mg $day^{-1}$ days 1–21 every 28 days were assumed to be standard single drug regimens.

whereas the anti-cancer effect was attenuated when topoisomerase II poisons were administered concurrently or immediately after topoisomerase inhibition (D'Arpa et al, 1990; Kaufmann, 1991; Bertrand et al, 1992; Kim et al, 1992).

These data led us to evaluate a chemotherapeutic regimen of topotecan in combination with etoposide, a representative inhibitor of topoisomerase II. We initiated a phase I and pharmacological study with topotecan and etoposide combined in a schedule that hypothetically maximizes the likelihood of producing synergistic effects while diminishing the likelihood of inhibitory effects, i.e. etoposide dosing preceded by topotecan infusion. Preclinical and clinical data indicate enhanced antineoplastic activity when topotecan is administered daily for prolonged periods of time. Most clinical responses were observed with a daily 30-min times five infusion schedule. The recommended dose for this schedule is 1.5 mg $m^{-2}$ $day^{-1}$ (Rowinsky et al, 1991; Verweij et al, 1993). Etoposide also exerts schedule dependency, with the greatest activity seen after prolonged exposure. As chronic infusion is cumbersome and expensive and anti-tumour activity is retained with repeated low administration for prolonged periods, an oral 'low-dose' formulation containing 20 mg of etoposide was developed in our hospital pharmacy (Jonkman-De Vries et al, 1996). The objectives of this phase I study were (a) to determine the maximum-tolerated doses for the combination of daily 30-min infusions of topotecan on days 1–5 followed by oral etoposide on days 6–12, (b) to characterize the dose-limiting toxicities associated with this regimen, and (c) to investigate the clinical pharmacology of topotecan and etoposide.

### PATIENTS AND METHODS

### Patient population

Patients were eligible if they had a histologically confirmed diagnosis of a solid malignant tumour that was not amenable to established forms of effective therapy. Other eligibility criteria included a Zubrod–ECOG–WHO performance status ≤ 2, anticipated life expectancy of ≥ 3 months and age ≥ 18 years. Previous anti-cancer chemotherapy had to be discontinued for at least 4 weeks before entry into the study or 6 weeks in cases of pretreatment with a nitrosourea or mitomycin C. All patients had acceptable bone marrow function white blood cell (WBC) $\geq 4 \times 10^9$ $l^{-1}$ and platelets $\geq 100 \times 10^9$ $l^{-1}$; normal hepatic function, serum bilirubin ≤ 1.5 mg $dl^{-1}$ (25 μmol $l^{-1}$) and other liver function tests ≤ twice the normal upper limit or ≤ five times when related to liver metastases, prothrombin or thrombotest within normal limits; normal renal function, serum creatinine ≤ 1.4 mg $dl^{-1}$ (120 μmol $l^{-1}$). The study protocol was approved by the Medical Ethics Committee of the hospital, and all patients gave written informed consent.

### Treatment plan and study design

Topotecan (SmithKline Beecham Pharmaceuticals, King of Prussia, PA, USA) was supplied by the National Cancer Institute as a lyophilized light yellow powder in vials containing 5 mg of topotecan (as the free base), 60 mg of mannitol, 25 mg of tartaric acid and 2 M hydrochloric acid and/or 0.05 M sodium hydroxide for pH adjustment to 3.0. The content of each vial was reconstituted with 2 ml of sterile water for injection, USP, yielding a 2.5 mg $ml^{-1}$ solution of topotecan. The appropriate dosage of the drug was diluted in 50 or 100 ml of 0.9% sodium chloride and was administered intravenously over 30 min by a syringe pump.

Etoposide capsules were manufactured at the hospital's pharmacy (Jonkman-de Vries et al, 1996). Etoposide was synthesized by Omnichem and obtained from Pharmachemie (Haarlem, The Netherlands). The capsule was a hard gelatin capsule (no. 3; Spruyt-Hillen, Utrecht, The Netherlands), which contained 20 mg of etoposide and 60 mg of microcrystalline cellulose (Avicel, Brocacef, Maarssen, The Netherlands).

The starting doses of topotecan and etoposide were 0.5 mg $m^{-2}$ on days 1–5 and 20 mg twice daily on days 6–12 respectively. The regimen was intended to be repeated every 4 weeks. This could be changed during the study to either shorter or longer intervals depending on bone marrow recovery. Doses were escalated in an alternating way according to the scheme in Table 1. Upon identification of the MTD, the study was designed to reduce the topotecan dose by one level and prolong etoposide dosing to 10 days. Treatment cycles were to be repeated at 3-week intervals. Patients were scheduled to receive at least two courses. No dose escalation was permitted in individual patients. Patients with progressive disease were removed from the study.

### Patient evaluation

Pretreatment evaluation included a complete medical history and complete physical examination. Before each course, chemistry and haematology profiles, creatinine clearance and urine were checked, as were chest radiographs and electrocardiograms. Haematology was checked twice weekly. Weekly evaluations

© Cancer Research Campaign 1997

included serum chemistry screen and urinalysis. Tumour measurements were performed every other cycle. All toxicities observed were graded according to the common toxicity criteria (CTC). The dose-limiting toxicity was defined as (a) grade IV neutropenia or thrombocytopenia for more than 7 days or neutropenic fever requiring hospitalization and i.v. antibiotics, (b) the impossibility to re-treat a patient according to schedule, or (c) other grade III–IV toxicities with the exception of alopecia, nausea and vomiting. The MTD was defined as the highest dose that can be safely administered to a patient producing tolerable, manageable and reversible toxicity of CTC grade III in at least two out of a maximum of six patients.

### Pharmacokinetic studies

Clinical pharmacokinetic studies of topotecan and etoposide were performed in at least two patients per dose level. On day 1, blood samples (5 ml each), taken from an in-dwelling intravenous cannula placed in the arm contralateral to the arm receiving topotecan, were collected in heparinized tubes at 11 time points: preinfusion at 5, 15 and 30 min during the infusion, and at 15 and 30 min and 1, 2, 3, 4, and 6 h after stopping the infusion. On day 6, patients received the morning dose (one or two capsules) of etoposide, and blood samples were taken before dosing and at 15, 30 and 45 min, and 1, 1.5, 2, 3, 4, 8, 12 and 24 h after ingestion. Additional etoposide blood samples were taken on day 12 during the first and subsequent courses to assess steady-state concentrations and possible accumulation. Plasma was obtained by immediate centrifugation of the samples (5 min; 3000 r.p.m.) and stored at –30°C until analysis. Urine was collected as 24-h aliquots from the start of infusion on days 1–5 and samples were frozen until analysis.

**Table 2** Patient characteristics

| | Number of patients | Response | | | |
|---|---|---|---|---|---|
| | | PR | SD | PD | NA |
| Number of patients | 30 | | | | |
| Male/female | 11/19 | | | | |
| Median age (range) | 54 (32–73) | | | | |
| Performance status | | | | | |
| 0 | 15 | | | | |
| 1 | 10 | | | | |
| 2 | 5 | | | | |
| Prior therapy | | | | | |
| None | 3 | | | | |
| Chemotherapy | 21 | | | | |
| Radiotherapy | 0 | | | | |
| Chemotherapy and radiotherapy | 6 | | | | |
| Tumour sites | | | | | |
| Ovary | 14 | 1 | 6 | 4 | 3 |
| Colorectal | 7 | | 3 | 3 | 1 |
| Lung (SCLC) | 2 | | 1 | | 1 |
| Gall bladder | 1 | | 1 | | |
| Stomach | 1 | | | 1 | |
| Pancreas | 1 | | 1 | | |
| Sarcoma | 1 | | 1 | | |
| Head/neck | 1 | | 1 | | |
| Fallopian tube | 1 | | 1 | | |
| Unknown origin | 1 | | 1 | | |

Abbreviations: SCLC, small-cell lung cancer; PR, partial response; SD, stable disease; PD, progressive disease; NA, not assessable

Plasma levels of total topotecan (lactone plus carboxylate form) were determined by a validated high-performance liquid chromatography (HPLC) method as developed in our laboratory (Rosing et al, 1995). Urine was diluted (1:50) with methanol, acidified with perchloric acid 2% (1:1) to a pH of 1.0, and directly injected onto the HPLC column. Plasma levels of etoposide were determined by HPLC using a modification of the method by Sinkule and Evans (1984). To 500 µl of plasma, 2 ml of 1,2-dichloro ethane and 100 µl of a 15 nmol ml$^{-1}$ stock solution of teniposide (used as an internal standard) were added. The sample was vortex-mixed for 10 s and then centrifuged for 10 min at 3000 r.p.m. The plasma layer was removed and the organic layer was evaporated under a nitrogen stream. The residue was reconstituted with 200 µl of 0.01 M phosphate buffer solution pH 6.5/acetonitrile (1:1, v/v) and aliquots of 50 µl were injected onto the HPLC column. Separation of the sample was accomplished by reverse-phase HPLC, using a Model SP8875 autosampler [Thermo Separation Products (TSP), Fremont, CA, USA], a Model 510 solvent delivery system (Waters Assoc., Milford, MA, USA), equipped with a µ-Bondapak phenyl analytical column (300 mm × 3.9 mm i.d.; particle size 10 µm; Waters Assoc.). The mobile phase consisted of water–acetonitrile–acetic acid (690:300:10, v/v/v). The flow rate was 1.0 ml min$^{-1}$ and analyses were performed at ambient temperature. Detection was performed at 281 nm using a Model Spectra 200 programmable wavelength detector (TSP). Quantitative computations were based on the ratio of the peak areas of etoposide and teniposide.

For either drug, the maximum plasma concentration after a single dose ($C_{max}$), the time to reach the maximum concentration ($T_{max}$) and the lag-time ($t_{lag}$) were determined graphically. The AUC was determined using the linear logarithmic trapezoidal method with extrapolation to infinity. Total body clearance from plasma (CL), apparent volume of distribution at steady-state ($V_{ss}$), elimination rate constant ($k$), elimination half-life ($t_{1/2}\beta$), and maximal and minimal steady-state concentrations ($C_{max,ss}$ and $C_{min,ss}$) of etoposide were calculated using standard equations (Rowland and Tozer, 1989).

Relationships between systemic exposure to either drug and pharmacodynamics, in particular the dose-limiting toxicities, were explored using scatter plots of the dose or pharmacokinetic parameters vs the percentage decrease in WBC, absolute neutrophil count (ANC) and platelets. The percentage decrease is defined as:

$$\%\text{ decrease} = \frac{\text{Pretreatment value} - \text{value of the nadir}}{\text{Pretreatment value}} \times 100\%$$

The data were fitted using a sigmoidal maximum effect ($E_{max}$) model, as described by the modified Hill equation (Holford and Sheiner, 1982):

$$E = \frac{(E_{max})(\text{DE})^{\gamma}}{(\text{DE}_{50})^{\gamma} + (\text{DE})^{\gamma}}$$

where E represents the observed effect (i.e. % decrease) observed at drug exposure DE, $E_{max}$ denotes the maximal elicitable effect, which is fixed at 100 (i.e. 100% decrease) for cytotoxicity. DE is a measure of drug exposure (i.e. AUC, $C_{max}$, time above a threshold concentration of 10 nmol l$^{-1}$ ($t$ > 10 nmol l$^{-1}$)), DE$_{50}$ represents the drug exposure associated with 50% of $E_{max}$ and $\gamma$ is the Hill coefficient, which describes the sigmoidity of the curve. Statistical analysis was performed with the Number Cruncher Statistical System (NCSS, Kaysville, UH, USA, 1992). Data are presented as means ± s.d.

© Cancer Research Campaign 1997

**Table 3** Haematological toxicity

| Dose level | Number of patients | Number of courses | Neutropenia | | Thrombocytopenia | | Anaemia | |
|---|---|---|---|---|---|---|---|---|
| | | | Grade 3 | Grade 4 | Grade 3 | Grade 4 | Grade 3 | Grade 4 |
| 1 | 3 | 22 | 0/0* | 0/0 | 0/0 | 0/0 | 0/2 | 0/1 |
| 2 | 4 | 16 | 2/4 | 0/1 | 2/4 | 0/0 | 0/2 | 0/0 |
| 3 | 3 | 12 | 1/3 | 0/1 | 0/0 | 0/0 | 0/0 | 0/0 |
| 4 | 6 | 20 | 4/6 | 3/6 | 1/1 | 1/1 | 0/1 | 1/1 |
| 3a | 7 | 34 | 1/6 | 3/13 | 2/5 | 0/0 | 2/3 | 0/0 |
| 2a | 6 | 27 | 3/12 | 2/4 | 1/3 | 0/0 | 0/0 | 0/0 |

*Number of patients developing toxicity in the first course/number of courses causing toxicity for all courses.

**Table 4** Nadir blood counts

| Dose level | Patients | Courses | ANC ($\times 10^9$ l$^{-1}$) | | Platelets ($\times 10^9$ l$^{-1}$) | | Haemoglobin (g dl$^{-1}$) | |
|---|---|---|---|---|---|---|---|---|
| | | | First course | All courses | First course | All courses | First course | All courses |
| 1 | 3 | 22 | 3.1 (1.4–3.4) | 2.0 (1.4–5.2) | 264 (203–344) | 211 (140–366) | 6.6 (5.8–8.1) | 5.9 (3.1–8.1) |
| 2 | 4 | 16 | 1.0 (0.5–1.3) | 1.1 (0.5–3.0) | 58 (33–82) | 66 (30–120) | 5.8 (5.0–7.5) | 6.1 (4.7–7.5) |
| 3 | 3 | 12 | 1.0 (0.5–1.5) | 1.2 (0.4–2.2) | 104 (97–195) | 175 (91–250) | 6.3 (5.9–6.9) | 5.7 (5.0–6.9) |
| 4 | 6 | 20 | 0.3 (0.0–0.8) | 0.7 (0.0–2.0) | 107 (8–187) | 164 (8–217) | 5.5 (4.1–7.4) | 6.8 (4.1–7.4) |
| 3a | 7 | 34 | 0.8 (0.1–1.3) | 0.8 (0.1–3.2) | 107 (31–268) | 86 (30–378) | 6.1 (4.4–7.8) | 5.9 (4.4–7.8) |
| 2a | 6 | 27 | 0.7 (0.4–1.0) | 0.8 (0.4–1.6) | 68 (31–264) | 202 (31–323) | 6.5 (4.9–7.5) | 6.2 (4.9–7.5) |

Numbers in parentheses show range of nadir values.

### RESULTS

A total of 30 patients was registered onto this trial. Eleven patients were men and 19 were women, with a median age of 54 years (range, 32–73). Twenty-five patients (83%) had a performance status of either 0 or 1. Diagnoses included a predominance of ovarian and colon cancers. All but three patients had received previous cytotoxic therapies. Thirteen and fourteen patients received one and two or more (median 3, range 2–4) previous chemotherapeutic regimens respectively. Additional patient characteristics are outlined in Table 2. A total of 131 full courses was administered, with a median number of four (range, 1–10) per patient. Four patients received only one course. In two patients, this was because of rapid progressive disease. One patient had a rapid deterioration in performance status immediately after registration onto the study and went off-study because of poor condition and unacceptable pre-existing vomiting. One patient developed grade IV neutropenia with fever, most probably atypical pneumonia, with negative blood cultures and fever persisting while on antibiotics. Of all courses administered at dose levels 1–4, two courses (4%) had to be postponed for 1 week because of unresolved neutropenia and fever, and a treatment delay of 2 weeks was required in one course because of an abscess and rapidly increasing ascitic fluid production. With the adjusted 3-week schedule (dose levels 2a and 3a, Table 1), only 56% of all treatment courses could be given at the planned interval because of unrecovered bone marrow. However, most patients (88%) could be re-treated by day 28. No intrapatient dose reductions or escalations were made.

Twenty-nine patients were assessable for toxicity during the first course. One patient was not evaluable because she received only topotecan. Her first course was abrogated because of rapid worsening of her performance status after registration onto the trial. Myelosuppression, primarily neutropenia, was the dose-limiting toxicity for the combination of topotecan and etoposide in this schedule (Table 3). Cohorts of three patients were entered and doses were escalated to topotecan 1.25 mg m$^{-2}$ and etoposide 40 mg twice daily (dose level 4). At this dose level DLT occurred in two out of six patients. One patient experienced grade IV neutropenia and grade III mucositis, another patient developed grade IV neutropenia with neutropenic fever. Both patients were heavily pretreated. The first patient had received three previous regimens for the treatment of metastasized SCLC, the second patient was an ovarian cancer patient who had received four previous regimens, including platinum-based therapy. To investigate further dose intensification, additional patients were treated at 3-week intervals with the topotecan dose reduced by one level to 1.0 mg m$^{-2}$, but with etoposide administration prolonged from 7 to 10 days. A time delay to retreatment emerged as DLT. In three out of seven patients, treatment was postponed for one week because of unrecovered bone marrow. One patient developed neutropenic fever. The etoposide dose was then reduced by one level to 20 mg twice daily. At this dose level, three out of six patients developed DLT. Two patients had treatment delays of one week because of unresolved neutropenia. One patient experienced grade III neurological toxicity, being motor function loss of the right hand. Pathology was non-diagnostic. Further attempts to dose intensification were not performed and the study was closed.

### Haematological toxicity

The principal toxicity was myelosuppression, primarily neutropenia. Table 4 lists the median and range of nadir haematological toxicity at each dose level during the first course and all

© Cancer Research Campaign 1997

**Figure 1** Representative plasma concentration vs time curves of topotecan as the sum of the lactone and carboxylate forms (—) and *N*-desmethyltopotecan (- - -) after a 30-min intravenous infusion of topotecan 0.5 mg m$^{-2}$ (■), 1.0 mg m$^{-2}$ (▲) and 1.25 mg m$^{-2}$ (●). The arrow indicates the end of infusion courses. The neutrophil nadir occurred on day 16 (range 9–24) for the initial courses, and on day 13 (range 8–24) for all courses. In the first course, the median duration of neutropenia was 9 days (range 3–17), with recovery to grade I on day 24 (range 16–29). At the first dose level, no grade III or IV haematological toxicity occurred. At the second dose level, four episodes of brief grade III neutropenia and one episode of grade IV neutropenia were experienced by two patients, including one heavily pretreated patient. At the third dose level, grade III and IV neutropenia occurred in 25% and 8% of courses respectively. At the fourth dose level, a higher proportion of courses was associated with grade IV neutropenia. Both grade III and IV neutropenia developed in 30% of all courses. When the protocol was amended to 10 days' etoposide administration in an effort to obtain higher dose intensity, relatively more patients experienced grade IV neutropenia. At dose level 3a, which is identical to dose level 3 except for the length of etoposide administration (10 and 7 days respectively), grade III and IV neutropenia was noted in 12% and 56% of courses respectively. The evaluation of nadir neutrophil and platelet counts in patients who received two or more courses showed no evidence of cumulative haematological toxicity.

Thrombocytopenia occurred less frequently than neutropenia, with only one episode of grade IV thrombocytopenia occurring in a patient at the fourth dose level, whereas 25, 5, 15 and 11% of courses at dose level 2, 4, 3a and 2a respectively, were associated with grade III thrombocytopenia. All but three patients experienced anaemia, which was severe (grade III and IV) in ten courses (8%), requiring the transfusion of a total of 72 U of packed red blood cells (RBCs) during 26 courses involving 14 patients.

### Non-haematological toxicities

Non-haematological drug toxicities were relatively mild. Transient grade I and II nausea and vomiting occurred in 38 and 19% of assessable courses, respectively, and could generally be controlled by standard antiemetics. Other grade I or II gastrointestinal side-effects

**Table 5** Pharmacokinetic parameters of total (lactone plus carboxylate) topotecan following a 30-min infusion

| Dose (mg m$^{-2}$ | Number of patients | $C_{max}$ (nmol l$^{-1}$) | $AUC_{0\to\infty}$ (h nmol$^{-1}$ l$^{-1}$) | CL (l h$^{-1}$ m$^{-2}$) | $V_{ss}$ (l m$^{-2}$) | $t_{½}\beta$ (h) |
|---|---|---|---|---|---|---|
| 0.5 | 3 | 48.9 ± 4.3 | 80.3 ± 10.2 | 14.9 ± 1.8 | 34.0 ± 2.6 | 1.9 ± 0.1 |
| 1.0 | 18 | 81.9 ± 21.6 | 184.6 ± 38.1 | 13.4 ± 2.6 | 36.4 ± 5.2 | 2.2 ± 0.3 |
| 1.25 | 3 | 104.8 ± 8.3 | 199.8 ± 51.2 | 15.6 ± 4.0 | 33.7 ± 6.7 | 1.8 ± 0.1 |

Abbreviations: see Materials and methods section.

**Table 6** Pharmacokinetic parameters of *N*-desmethyltopotecan after a 30-min infusion of topotecan

| Dose (mg m$^{-2}$) | Number of patients | $C_{max}$ (nmol l$^{-1}$) | $AUC(t)_{0\to6.5\,h}$ (h nmol$^{-1}$ l$^{-1}$) | AUC(*t*) ratio (%) | $t_{max}$ (h) | $t_{lag}$ (h) |
|---|---|---|---|---|---|---|
| 0.5 | 3 | 0.23 ± 0.10 | 1.04 ± 0.43 | 1.3 ± 0.6 | 4.2 ± 0.6 | 0.7 ± 0.2 |
| 1.0 | 18 | 0.43 ± 0.21 | 2.05 ± 1.09 | 1.1 ± 0.5 | 3.4 ± 1.1 | 0.6 ± 0.2 |
| 1.25 | 3 | 0.53 ± 0.34 | 2.60 ± 1.74 | 1.4 ± 0.9 | 2.9 ± 0.5 | 0.6 ± 0.4 |

Abbreviations: see Materials and methods section; $AUC(t)_{0\to6.5\,h}$, area under the plasma concentration vs time curve up to last measured point; AUC(*t*) ratio, metabolite-to-topotecan AUC(*t*) ratio.

**Table 7** Pharmacokinetic parameters of etoposide after oral administration

| Dose (mg) | Number of patients | $C_{max}$ (µmol l$^{-1}$) | $t_{max}$ (h) | $AUC_{0\to\infty}$ (h µmol$^{-1}$ l$^{-1}$) | $t_{½}\beta$ (h) | $C_{max,ss}$ (µmol l$^{-1}$) | $C_{min,ss}$ (µmol l$^{-1}$) |
|---|---|---|---|---|---|---|---|
| 20 | 7 | 0.75 ± 0.54 | 2.4 ± 1.2 | 5.39 ± 2.88 | 4.8 ± 2.5 | 1.02 ± 0.49 | 0.17 ± 0.15 |
| 40 | 8 | 1.23 ± 0.57 | 2.3 ± 1.0 | 12.97 ± 8.51 | 7.8 ± 5.3 | 1.82 ± 0.83 | 0.61 ± 0.65 |

Abbreviations: see Materials and methods section.

© Cancer Research Campaign 1997

**Figure 2** The percentage decrease in ANC vs topotecan AUC during course 1 (■, —). The curve is fitted to a sigmoidal $E_{max}$ model (parameters: $AUC_{50}$ = 79.2 h nmol$^{-1}$ l$^{-1}$; $\gamma$ = 2.1). These data are superimposed on the sigmoidal model derived from a preceding single-agent phase I trial (□, - -; $AUC_{50}$ = 79.2 h nmol$^{-1}$ l$^{-1}$; $\gamma$ = 1.8) (Van Warmerdam et al, 1995)

included mucositis (11% of courses; six patients), being severe (grade III) in one patient at the fourth dose level, diarrhoea (17% of courses; 11 patients), obstipation (15% of courses; eight patients), and mouth dryness (7% of courses; three patients). Alopecia was observed in 19 patients, and was complete in five patients. Six patients experienced neurotoxicity during 18 courses (14%) at all dose levels, being severe (grade III) in one patient at dose level 2a, who experienced motor function loss of the right hand for several days during his first course. Neurotoxicity primarily manifested in tingling hands and toes and was pre-existing in three patients who had been previously treated with platinum-based regimens. One ovarian cancer patient at dose level 2a had grade I cystitis for 10 days, which was possibly drug-related. Four patients (5% of courses) developed microscopic haematuria (grade I) and eight patients (15% of courses) had mild proteinuria. The most common complaint was fatigue (35% of courses). All toxicities were reversible and not dose-related.

### Pharmacokinetics

Twenty-four patients underwent pharmacokinetic monitoring on day 1 of topotecan administration of the first course. Fifteen patients also had blood samples taken with their first dose of etoposide (day 6). Representative plasma concentration vs time curves of total topotecan are shown in Figure 1. Mean topotecan $C_{max}$ and AUC increased linearly with dose in the dose range used, but there was considerable overlap in individual values between different dose levels. Individual elimination curves of topotecan were well described using conventional compartmental modelling. A two-compartment model provided the best fit to the data, based on visual inspection and Akaike's information criteria (AIC). Post-infusion plasma levels declined in a biphasic manner with mean alpha and beta half-lives of 3.8 ± 3.2 min and 2.1 ± 0.3 h respectively. The systemic clearance rate for topotecan was 13.8 ± 2.7 l h$^{-1}$ m$^{-2}$ and the steady-state volume of distribution was 36.7 ± 6.2 l m$^{-2}$. Pharmacokinetic parameters are presented in Table 5. Two patients at the 1.0 mg m$^{-2}$ dose level had ascitic fluid analysed for topotecan. Ascitic fluid concentrations of 9.51 and 11.63 nmol l$^{-1}$ were obtained at approximately 2 h after infusion, with ascitic fluid-to-plasma ratios of 0.54 and 0.52. Topotecan 24-h urinary excretion data over 5 days were available for 27 patients. The percentage of the administered dose recovered in the urine was 41 ± 10% (range 25–71).

During HPLC analysis of plasma and urine samples, an unidentified peak eluting just after topotecan was observed in the chromatograms, which was not present in blank and calibration samples. The compound was isolated from urine and identified to be as the *N*-desmethyl derivative of topotecan (Rosing et al, 1997). Under our assay conditions topotecan and *N*-desmethyltopotecan had equivalent extraction ratios and fluorescence yields that permitted metabolite levels to be quantified using the topotecan calibration curve. Desmethyltopotecan appeared in plasma after cessation of the 30-min topotecan infusion, with a lag-time of 0.58 ± 0.24 h. Maximal metabolite plasma concentrations of 0.23 ± 0.10, 0.43 ± 0.21 and 0.53 ± 0.34 nmol l$^{-1}$ were obtained after respective topotecan doses of 0.5, 1.0 and 1.25 mg m$^{-2}$ (Figure 1 and Table 6). The time to reach maximal plasma levels was 3.4 ± 1.0 h. The metabolite-to-topotecan ratio of maximal plasma levels was 0.5%. At the last measured time point (6 h post-infusion) desmethyltopotecan plasma levels were still at or near the maximal concentration. The AUC(t) was calculated up to this last measured time point. The metabolite-to-topotecan AUC(t) ratio was 0.012. Plasma levels of desmethyltopotecan at 2 h after infusion on days 2 to 5 were significantly higher than on day 1, ranging from 42 ± 26% higher on day 2 to 53 ± 41% on day 5, which suggests accumulation of this species. The 24-h urinary recovery of desmethyltopotecan was 1.4 ± 0.6% of the administered topotecan dose. In four patients, urine was collected for 48 h after the fifth daily topotecan infusion. An additional 0.4% was excreted in the urine as the metabolite from 24 to 48 hours after infusion.

Etoposide pharmacokinetics were described using non-compartmental modelling techniques. Maximum plasma levels of 0.75 ± 0.54 and 1.23 ± 0.57 μmol l$^{-1}$ were reached at 2.4 ± 1.2 and 2.3 ± 1.0 h after ingestion of respective etoposide single doses of 20 mg and 40 mg (Table 7). The maximum steady-state plasma concentrations were calculated as 1.02 ± 0.49 and 1.82 ± 0.83 μmol l$^{-1}$ after doses of 20 and 40 mg twice daily respectively. The plateau trough concentrations were calculated as 0.17 ± 0.15 and 0.61 ± 0.65 μmol l$^{-1}$ respectively. No drug accumulation could be detected from repetitive determination of steady-state etoposide concentrations during the first and subsequent courses. The mean plasma concentrations at day 12 of all courses were 0.71 ± 0.56 μmol l$^{-1}$ ($n$ = 8) after 20 mg twice daily, and 1.78 ± 0.61 μmol l$^{-1}$ ($n$ = 24) after 40 mg twice daily, which are within the range of calculated maximal and minimal steady-state levels.

### Pharmacodynamics

The relationships between topotecan pharmacokinetic parameters (i.e. AUC, $t$ > 10 nmol l$^{-1}$, $C_{max}$) or dose and myelosuppression during the first course could be adequately described by a sigmoidal $E_{max}$ model. Topotecan AUC was most predictive of the percentage of decrease in WBC ($r^2$ = 0.70) and ANC ($r^2$ = 0.65). Interestingly, the latter relationship could be superimposed on the model derived from a preceding phase I trial of single-agent topotecan (Van Warmerdam et al, 1995). A plot of this relationship is depicted in Figure 2. $T$ > 10 nmol l$^{-1}$ and dose also correlated well with the percentage of decrease in neutrophils ($r^2$ = 0.60 in both cases). With this model, the dose associated with a 50% decrease in ANC ($D_{50}$) was 0.6 mg m$^{-2}$ day$^{-1}$. In all cases, the

© Cancer Research Campaign 1997

sigmoidal model produced better fits compared with linear equations. Desmethyltopotecan AUC(t) was not related to myelosuppression. In addition, exploring correlations between etoposide pharmacokinetics (i.e. AUC, $C_{max}$) or total dose and haematological toxicity did not yield any significant relationship.

### Responses

Twenty-four patients were assessable for therapeutic efficacy. A partial response occurred in a 55-year-old woman with ovarian cancer. Her response was documented after four courses of topotecan 1.0 mg $m^{-2}$ $day^{-1}$ for 5 days and etoposide 40 mg twice daily for 10 days (dose level 3a) and was brief (3 months). Sixteen patients (64%) had stable disease for at least 4 months (range 4–10+). Four ovarian cancer patients had significantly decreased tumour markers (CA-125) to normal values, but remained stable.

### DISCUSSION

Preclinical studies have demonstrated mixed results when topoisomerase I and II inhibitors were combined. The reason for the discrepancies in cytotoxic effects of the combination might relate to the scheduling of both kinds of inhibitors, the cancer cell lines and the camptothecin analogue used. Additive and synergistic effects were observed for simultaneous exposure to etoposide and CPT-11 on acute lymphoblastic leukaemia cells and human lung cancer cell lines (Kano et al, 1992; Takada et al, 1992) Contrary to these results, simultaneous treatment of hamster lung fibroblasts (D'Arpa et al, 1990), human leukaemia cells (Kaufmann, 1991), human colon carcinoma cells (Bertrand et al, 1992), and human tumour xenografts (Kim et al, 1992) with camptothecin analogues and etoposide or doxorubicin attenuated the cytotoxicity of these agents over single-agent exposure. Sequential administration of camptothecins and etoposide was shown to circumvent the antagonistic cytotoxicity (Bertrand et al, 1992; Chang et al, 1992; Cheng et al, 1994). Bertrand et al (1992) reported additive enhancement of cytotoxicity in human colon carcinoma cells, provided camptothecin and etoposide were administered 6–8 h apart. The additive effect did not depend on the order of administration. However, several authors emphasized a sequence dependency in the cytotoxic effect. Kozelsky et al (1995) showed maximum synergy in hamster lung fibroblast cells with etoposide administration after topotecan exposure compared with the opposite sequence. Masumoto et al (1995) demonstrated that significant synergy occurred only when etoposide was given after a 48-h drug-free interval after treatment with SN-38, the active metabolite of CPT-11, whereas cytotoxicity was slightly reduced when etoposide was administered concurrently with or immediately after SN-38. CPT-11 pretreatment was shown to enhance the cytotoxicity of doxorubicin by increasing topoisomerase-II mRNA expression and the S-phase cell population 24 and 48 h after CPT-11 treatment, respectively, in the case of oesophageal and colon tumour xenografts (Kim et al, 1992).

We performed a dose-finding study of topotecan combined with etoposide in a sequential fashion. A suitable dose for use in phase II clinical trials is topotecan 1.0 mg $m^{-2}$ on days 1–5 and etoposide 40 mg twice daily on days 6–12 every 4 weeks. Neutropenia was the principal dose-limiting toxicity of this combination. The relative low dose intensity of etoposide at the recommended dosage (Table 1) led us to focus on extended etoposide dosing from 7 to 10 days. Concurrently, we attempted to reduce the drug-free interval, as stabilization of the topoisomerase enzyme–DNA complex by topoisomerase inhibitors is a readily reversible process. With the removal of the drug, enzyme activity is restored and DNA damage repaired. However, dose intensification with treatment at a 3-week cycle and protracted etoposide administration was not feasible. With this adjusted schedule, only 56% of all treatment courses could be given at the planned 3-week interval because of unrecovered bone marrow, but most patients (88%) could be re-treated by day 28. The definition of DLT in this protocol (time delay to retreatment) results in our recommendation of dose level 3 for phase II trials. However, based on observed complete recovery from neutropenia on day 28 and the absence of cumulative myelotoxicity, treatment at doses of topotecan 1.0 mg $m^{-2}$ on days 1–5 and etoposide 40 mg twice daily on days 6–15 (dose level 3a) should be possible in 4-week cycles to obtain a higher dose intensity. At these doses, 68% of courses was associated with grade III or IV neutropenia. A comparison of the current study with single-agent phase I studies shows that topotecan in combination with prolonged low-dose oral etoposide could only be given at 67–83% of the single-agent dosage. Rowinsky et al (1991) and Verweij et al (1993) recommended a dose of 1.5 mg $m^{-2}$ $day^{-1}$ for single-agent topotecan in the 30-min daily times five infusion schedule repeated every 3 weeks for both minimally and heavily pretreated patients. At this dose, approximately 75% of courses resulted in grades III or IV leucocytopenia and neutropenia (Rowinsky et al, 1991; Verweij et al, 1993). Saltz et al (1993) recommended doses of 1.5 and 1.25 mg $m^{-2}$ daily in previously untreated and previously treated patients respectively. This indicates that bone marrow suppression of the combination is more severe compared with topotecan administered singly. Similar results have been reported previously. A combination regimen of topotecan administered as a 72-h infusion followed by bolus doxorubicin on day 5 resulted in unexpected severe neutropenia at the first dose level (topotecan/doxorubicin 0.35/45 mg $m^{-2}$ $day^{-1}$) (Tolcher et al, 1994). Further dose escalation was performed with G-CSF support. Another study investigated a 72-h continuous infusion of topotecan, dose range 0.17–1.05 mg $m^{-2}$ followed by etoposide 100 mg $m^{-2}$ given over 2 h on days 7, 8 and 9 (Eckardt et al, 1993) Haematological toxicities were more severe than expected from singly administered topotecan and etoposide, which was suggested to represent a synergistic effect of the combination. Topoisomerase II levels were found to be markedly increased immediately before etoposide administration on day 7, and were decreased on day 9 (Eckardt et al, 1994).

In the current trial, the sequential combination of topotecan and etoposide induced minimal platelet count depression, with grade III to IV thrombocytopenia occurring in 11% of all courses. Anaemia requiring RBC transfusions occurred in 20% of all courses involving 14 patients. More modest anaemia was noted in most courses. The incidence of non-haematological adverse effects was typical for topotecan. Mild to moderate nausea and fatigue were most troublesome. Reversible neurotoxicity occurred in six patients, being severe (grade III) in one patient at dose level 2a, and was thought to be platinum-related in three patients. Neurotoxicity has not been noted frequently with either topotecan or etoposide. One patient developed mild cystitis (grade I) during her seventh course. Haemorrhagic cystitis was a major toxicity encountered with sodium camptothecin, but has not been observed with topotecan (Rowinsky et al, 1991; Verweij et al, 1993). Except for incidental findings of mild proteinuria and microscopic haematuria, no other episodes of renal toxicity were observed in this study.

© Cancer Research Campaign 1997

Topotecan pharmacokinetic parameters were similar to those reported in previous pharmacokinetic studies (Herben et al, 1996). The current study has used total (lactone plus carboxylate form) topotecan levels in determining drug exposure. The lactone form of topotecan, which is the active cytotoxic form, is quite labile and undergoes a rapid pH-dependent reversible hydrolysis to a carboxylated open-ring form under physiological conditions. Although the carboxylate form lacks topoisomerase inhibiting activity, the potential for conversion to the lactone suggests that cytotoxic activity should be possible regardless of what proportion of topotecan is in the open-ring form in the extracellular space. Previous studies have demonstrated that total topotecan AUC and $C_{max}$ values were equally, or even better, related to haematological toxicity compared with lactone AUC or $C_{max}$. This finding has led several authors to question the need for all efforts to quantitate lactone levels separately (Grochow et al, 1992; Stewart et al, 1994).

This is the first study reporting the pharmacokinetics of a metabolite of topotecan. During HPLC analysis of topotecan plasma and urine samples, we became aware of an unidentified peak eluting just after topotecan that was not present in preinfusion samples. The potential metabolite was isolated from urine samples (Rosing et al, 1997). Mass spectrometry data, along with HPLC retention and fluorescence data, demonstrated that this compound was identical to a synthetic reference standard *N*-desmethyltopotecan (SB 209780) (Boehm et al, 1991). Like topotecan, the desmethyl derivative possesses a lactone ring that is reversibly hydrolysed to an open carboxylate form (Rosing et al, 1997); it has slightly less anti-tumour activity compared with the parent compound (Johnson and Wood, personal communication). The metabolite appeared in plasma after cessation of the 30-min topotecan infusion. Maximal plasma levels were reached at approximately 3 h after infusion. Desmethyltopotecan was found to accumulate during 5 consecutive days of treatment. In view of the relative low amounts found in plasma, the clinical importance of the metabolite seems limited.

Etoposide pharmacokinetic parameters were similar to those reported in a preceding bioavailability study of the etoposide capsules used in the current trial (Jonkman-de Vries et al, 1996). This indicates that topotecan does not interfere with etoposide pharmacokinetics. In view of extrapolation of the results of this trial to future studies, it is important to mention that the bioavailability of the etoposide capsules used in this study was approximately 34%, which is lower than the normal value of around 70% of the commercially available etoposide capsules (Vepesid; 50 mg). In addition, it appeared that the home-made capsules acted, to some extent, as slow-release devices. The terminal half-life of the drug was significantly higher after oral than after intravenous administration (Jonkman-de Vries et al, 1996).

The severity of neutropenia was more closely related to the AUC of topotecan than to the AUC of its metabolite or etoposide. Patients who received equal topotecan doses but different dose intensities to etoposide, show the same sigmoidal relationship between topotecan dose or AUC and the percentage of decrease in ANC. The obtained model superimposed on the sigmoidal curve fit found in the phase I study of a daily times five infusion schedule of topotecan alone. It thus appears that in this schema the percentage decrease in ANC is primarily dependent on systemic exposure to topotecan. Nonetheless, a correlation coefficient of 0.81 ($r^2 = 0.65$) illustrates that the pharmacokinetic variability of topotecan is not the only determinant of this toxicity.

Conclusive evidence on the contribution of etoposide to the myelotoxicity is lacking. Etoposide-induced toxicity has been shown to be dose and schedule dependent. Prolonged exposure to plasma etoposide concentrations of around 1.7 $\mu$mol $l^{-1}$ has been advocated to be optimal for anti-tumour activity (Slevin et al, 1989; Thompson et al, 1993), whereas peak plasma concentrations above 17 $\mu$mol $l^{-1}$ are associated with significant toxicity (Clark et al, 1989). The maximal plasma concentrations obtained in this study are apparently below the threshold concentration needed to cause a marked decrease in neutrophils. This hypothesis is evidenced by results from the bioavailability study of the etoposide capsules used in this study, in which no episodes of neutropenia were observed among a total of 14 patients receiving 20 mg twice daily for prolonged periods (range 7–21 days) (Jonkman-de Vries et al, 1996).

In summary, topotecan and etoposide given in combination could not be administered at their individual MTDs. With combinations of myelosuppressive agents, it is difficult to determine whether the observed myelotoxic effect is significantly higher, i.e synergistic, or not statistically different, i.e. additive, compared with the effect that could theoretically be expected (Merlin et al, 1994). Modelling the toxic effects of a two-drug combination requires the relationship between pharmacokinetics and pharmacodynamics of each individual agent to be known. In this study, only the relationship between topotecan pharmacokinetics and resulting toxicity is known. The observed haematological toxicity, measured by the percentage decrease in neutrophil count, is not significantly different from the expected toxicity of topotecan given alone at the same dosage based on the previously documented concentration–effect relationship of topotecan. This implies that the sequential combination of topotecan and etoposide most likely results in additive, rather than synergistic myelosuppressive interaction. In view of the anti-tumour activity of this combination, only one brief partial response was observed in an ovarian cancer patient, whereas 64% of evaluable patients had stable disease. Phase II trials will be needed to establish the benefit of this combination over single-agent administration.

### REFERENCES

Ardizzoni A, Hansen HH, Dombernowsky P, Kaplan S, Postmus PE, Gamucci T, Schaefer B, Wanders J and Verweij J (1995) Phase II study of topotecan in refractory and sensitive small cell lung cancer (SCLC). *Eur J Cancer* **31A**: S19

Armstrong D, Rowinsky E, Donehower R, Rosenshein N, Walczak J and McGuire W (1995) Phase II trial of topotecan as salvage therapy in epithelial ovarian cancer. *Proc Am Soc Clin Oncol* **14**: 275

D'Arpa P and Liu LF (1989) Topoisomerase-targeting antitumor drugs. *Biochim Biophys Acta* **989**: 163–177

D'Arpa P, Beardmore C and Liu LF (1990) Involvement of nucleic acid synthesis in cell killing mechanisms of topoisomerase poisons. *Cancer Res* **50**: 6919–6924

Bertrand R, O'Connor PM, Kerrigan D and Pommier Y (1992) Sequential administration of camptothecin and etoposide circumvents the antagonistic cytotoxicity of simultaneous drug administration in slowly growing human colon carcinoma HT-29 cells. *Eur J Cancer* **28A**: 743–748

Boehm JC, Hecht SM and Holden KG (1991) Water-soluble camptothecin analogs. US patent no. 5,004,758

Chang JY, Dethlefsen LA, Barley LR, Zhou BS and Cheng YC (1992) Characterization of camptothecin-resistant Chinese hamster lung cells. *Biochem Pharmacol* **43**: 2443–2452

Chang AY, Garrow G, Boros L, Asbury R, Pandya K and Keng P (1995) Clinical and laboratory studies of topotecan in breast cancer. *Proc Am Soc Clin Oncol* **14**: 105

Cheng M-F, Chatterjee S and Berger NA (1994) Schedule-dependent cytotoxicity of topotecan alone and in combination chemotherapeutic regimens. *Oncol Res* **6**: 269–279

© Cancer Research Campaign 1997 

Clark PI, Joel SP and Slevin ML (1989) A pharmacokinetic hypothesis for the clinical efficacy of etoposide in small cell lung cancer. *Proc Am Ass Clin Oncol* **8**: 66

Cummings J and Smyth JF (1993) DNA topoisomerase I and II as targets for rational design of new anticancer drugs. *Ann Oncol* **4**: 533–543

DeVita VT. (1993) Principles of chemotherapy. In *Principles and practice of oncology*, 4th edn, DeVita VT, Hellman S and Rosenberg SA (eds), pp. 283–286, JB Lippincott: Philadelphia

Eckardt JR, Burris HA, Rodrigues GA, Fields SM, Rothenberg ML, Moore TD, Smith SC, Ganapathi R, Weiss GR, Johnson RK, Kuhn JG and Von Hoff DD (1993) A phase I study of the topoisomerase I and II inhibitors topotecan (T) and etoposide (E). *Proc Am Soc Clin Oncol* **12**: 137

Eckardt JR, Burris HA, Von Hoff DD, Rodriguez GI, Fields SM, Rothenberg ML, Moore TD, Hodges S, Weiss GR, Cobb P, Rinaldi D, Kuhn JG, Ford J and Ganapathi R (1994) Measurement of tumor topoisomerase I and II levels during the sequential administration of topotecan and etoposide. *Proc Am Soc Clin Oncol* **13**: 141

Grochow LB, Rowinsky EK, Johnson R, Ludeman S, Kaufmann SH, McCabe FL, Smith BR, Hurowitz L, DeLisa A, Donehower RC and Noe DA (1992) Pharmacokinetics and pharmacodynamics of topotecan in patients with advanced cancer. *Drug Metab Dispos* **20**: 706–713

Herben VMM, Ten Bokkel Huinink WW and Beijnen JH (1996) Clinical pharmacokinetics of topotecan. *Clin Pharmacokin* **31**: 85–102

Holford NHG and Sheiner LB (1982) Kinetics of pharmacologic response. *Pharmacol Ther* **16**: 143–166

Jonkman-De Vries JD, Herben VMM, Van Tellingen O, Dubbelman AC, Ten Bokkel Huinink WW, Rodenhuis S, Bult A and Beijnen JH (1996) Oral bioavailability of low-dose (20 mg) 'home-made' etoposide capsules. *Clin Drug Invest* **12**: 298–307

Kano Y, Suzuki K, Akutsu M and Suda K, Inoue Y, Yoshida M, Sakamoto S and Miura Y (1992) Effects of CPT-11 in combination with other anticancer agents in culture. *Int J Cancer* **50**: 604–610

Kaufmann SH (1991) Antagonism between camptothecin and topoisomerase II-directed chemotherapeutic agents in a human leukemia cell line. *Cancer Res* **51**: 1129–1136

Kim R, Hirabayashi N, Nishiyama M, Jinushi K, Toge T and Okada K (1992) Experimental studies on biochemical modulation targeting topoisomerase I and II in human tumor xenografts in nude mice. *Int J Cancer* **50**: 760–766

Kozelsky TK and Bonner JA (1995) Sequence dependent interaction of etoposide and topotecan. *Proc Am Ass Cancer Res* **36**: 293

Masumoto N, Nakano S, Esaki T, Tatsumoto T, Fujishima H, Baba E, Nakamura and Niho Y (1995) Sequence-dependent modulation of anticancer drug activities by 7-ethyl-10-hydroxycamptothecin in an HST-1 human squamous carcinoma cell line. *Anticancer Res* **15**: 405–410

Merlin J-L (1994) Concepts of synergism and antagonism. *Anticancer Res* **14**: 2315–2320

Pommier Y (1993) DNA topoisomerase I and II in cancer chemotherapy: update and perspectives. *Cancer Chemother Pharmacol* **32**: 103–108

Rosing H, Doyle E, Davies BE and Beijnen JH (1995) High-performance liquid chromatographic determination of the novel antitumour drug topotecan and topotecan as the total of the lactone plus carboxylate forms, in human plasma. *J Chrom B* **668**: 107–111

Rosing H, Herben VMM, Van Zomeren DM, Hop E, Kettenes-Van Den Bosch JJ, Ten Bokkel Huinink WW and Beijnen JH (1997) Isolation and structural confirmation of N-des-methyl topotecan, a metabolite of topotecan. *Cancer Chemother Pharmacol* (in press)

Rowinsky EK, Grochow LB, Hendriks CB, Ettinger DS, Forastiere AA, Hurowitz LA, McGuire WP, Sartorius SE, Lubejko BG, Kaufmann SH and Donehower RC (1991) Phase I and pharmacologic study of topotecan: a novel topoisomerase I inhibitor. *J Clin Oncol* **10**: 647–656

Rowland M and Tozer TN (1989) *Clinical Pharmacokinetics: Concepts and Applications*, 2nd edn, pp. 78–97 Lea and Febiger: Philadelphia

Saltz L, Sirott M, Young C, Tong W, Niedzwiecki D, Tzy-Jyun Y, Tao Y, Trochanowski B, Wright P, Barbosa K, Toomasi F and Kelsen D (1993) Phase I clinical and pharmacology study of topotecan given daily for 5 consecutive days to patients with advanced solid tumors, with attempt at dose intensification using recombinant granulocyte colony-stimulating factor. *J Natl Cancer Inst* **85**: 1499–1507

Sinkule JA and Evans WE (1984) High-performance liquid chromatographic analysis of the semisynthetic epipodophyllotoxins teniposide and etoposide using electrochemical detection. *J Pharm Sci* **73**: 164–168

Slevin ML, Clark, PI, Joel SP, Malik S, Osborne RJ, Gregory WM, Lowe DG, Reznek RH and Wrigley PF (1989) A randomized trial to evaluate the effect of schedule on the activity of etoposide in small cell lung cancer. *J Clin Oncol* **7**: 1333–1340

Stewart CF, Baker SD, Heideman RL, Jones D, Crom WR and Pratt CB (1994) Clinical pharmacodynamics of continuous infusion topotecan in children: systemic exposure predicts hematologic toxicity. *J Clin Oncol* **12**: 1946–1954

Sugimoto Y, Tsukahara S, Oh-Hara T, Liu LF and Tsuruo T (1990) Elevated expression of DNA topoisomerase II in camptothecin-resistant human tumor cell lines. *Cancer Res* **50**: 7962–7965

Takada M, Fukuoka M, Kudoh S, Masuda N, Nakagawa K, Kishimoto S (1992) Synergistic effects of CPT-11 and cisplatin or etoposide on human lung cancer cell lines and xenografts in nude mice. *Proc Am Ass Cancer Res* **33**: 226

Tan KB, Mattern MR, Eng W-K, McCabe FL and Johnson RK (1989) Nonproductive rearrangement of DNA topoisomerase I and II genes: Correlation with resistance to topoisomerase inhibitors. *J Natl Cancer Inst* **81**: 1732–1735

Thompson DS, Hainsworth JD, Hande KR, Holzmer MC and Greco FA (1993) Prolonged administration of low-dose, infusional etoposide in patients with etoposide-sensitive neoplasmas: a phase I/II study. *J Clin Oncol* **11**: 1322–1328

Tolcher AW, O'Shaughnessy JA, Weiss RB, Myhand RC, Schneider E, Hakim F, Noone M, Goldspiel B, Kohler D and Cowan KH (1994) A phase I study of topotecan (a topoisomerase I inhibitor) in combination with doxorubicin (a topoisomerase II inhibitor). *Proc Am Soc Clin Oncol* **13**: 157

Van Warmerdam LJC, Verweij J, Schellens JHM, Rosing H, Davies BE, De Boer-Dennert M, Maes RAA and Beijnen JH (1995) Pharmacokinetics and pharmacodynamics of topotecan administered daily for 5 days every 3 weeks. *Cancer Chemother Pharmacol* **35**: 237–245

Verweij J, Lund B, Beijnen J, Planting A, De Boer-Dennert M, Koier I, Rosing H and Hansen H (1993) Phase I and pharmacokinetics study of topotecan, a new topoisomerase I inhibitor. *Ann Oncol* **4**: 673–678

Wang JC (1985) DNA topoisomerases. *Annu Rev Biochem* **54**: 665–697

© Cancer Research Campaign 1997